# Regulatory Focus, Motivation, and Their Relationship With Creativity Among Adolescents

**DOI:** 10.3389/fpsyg.2021.666071

**Published:** 2021-05-20

**Authors:** Ling Wang, Yue Cui, Xinjing Wang, Jin Wang, Kaiye Du, Zheng Luo

**Affiliations:** ^1^Beijing Key Laboratory of Learning and Cognition, School of Psychology, Capital Normal University, Beijing, China; ^2^Beijing Xuanwu Foreign Language Experimental School, Beijing, China

**Keywords:** regulatory focus, intrinsic motivation, extrinsic motivation, creativity, mediator, adolescents

## Abstract

Due to the close relationship among intrinsic/extrinsic motivation, regulatory focus, and creativity revealed by previous literature, intrinsic/extrinsic motivation may play a mediating role between regulatory focus and creativity. Therefore, the present study aimed to investigate the relationship between regulatory focus and creativity by combining intrinsic/extrinsic motivation. In this study, senior high school students (*n* = 418) completed the Regulatory Focus Questionnaire, the Working Preference Inventory, the Williams Creativity Assessment Packet, and the Kirton Adaption–Innovation Inventory. The correlation analysis showed that both promotion and prevention focus positively correlated with intrinsic motivation; intrinsic motivation and promotion focus positively correlated with creativity personality and innovative-adaptive cognitive style; and extrinsic motivation and prevention focus negatively correlated with innovative–adaptive cognitive style. Furthermore, a path model showed that promotion focus positively predicted creativity through the mediation of intrinsic motivation. In general, our study suggests that intrinsic motivation plays a mediating role between promotion focus and creativity. Our results complement those of previous studies and serve as inspiration for the cultivation of creativity in classroom or enterprise settings.

## Introduction

Creativity plays an increasingly important role in environmental adaptation from both individual and organizational perspectives in the increasingly changing world. Previous studies have revealed that both regulatory focus and intrinsic/extrinsic motivation have a significant impact on creativity (Amabile, 1983; Guo et al., 2000; Eisenberger and Rhoades, 2001; Lam and Chiu, 2002; Herman and Reiter-Palmon, 2011; Bittner and Heidemeier, 2013; Sacramento et al., 2013; Tang et al., 2015); however, it remains unclear whether intrinsic/extrinsic motivation plays a mediating role between regulatory focus and creativity. Thus, the present study attempted to examine the relationship between regulatory focus and creativity in conjunction with intrinsic/extrinsic motivation.

The regulatory focus theory describes how individuals achieve goals through self-regulation (Higgins, 1997). Higgins (1997) distinguished between two kinds of regulatory focus, i.e., promotion focus and prevention focus; the former reflects an inclination toward hope, aspiration, and accomplishment, whereas the latter reflects an inclination toward security, safety, and responsibility. Individuals who are promotion focused pay more attention to information related to positive outcomes, and they prefer eager strategies to obtain gains; in contrast, individuals who are prevention focused usually concentrate more on information related to negative outcomes, and they prefer vigilant strategies to avoid possible losses (Higgins et al., 2001). The literature on the relationship between regulatory focus and creativity has revealed that promotion focus is positively correlated with creativity, while prevention focus is negatively correlated with creativity (Lam and Chiu, 2002; Herman and Reiter-Palmon, 2011; Bittner and Heidemeier, 2013; Sacramento et al., 2013). For example, Friedman and Förster (2001) found that compared with prevention focus, promotion focus is more conducive to producing creative thinking and creative ideas. Researchers have also found that promotion focus prompts people to seek more solutions during problem solving, thus, improving the fluency of idea generation in creative tasks (Lam and Chiu, 2002). Jin et al. (2016) reported that promotion focus could positively predict creativity, while prevention focus negatively predicted creativity. This relation may be explained by the impact of regulatory focus on attention and the cognitive process (Bittner and Heidemeier, 2013; Sacramento et al., 2013). Promotion focus can expand an individual’s attention and is conducive to the acquisition of more extensive cognitive elements, which benefits fluency and flexibility (Derryberry and Tucker, 1994; Förster et al., 2006). To sum up, promotion focus is positively associated with creativity, while prevention focus is negatively associated with creativity.

Intrinsic motivation is one of the most important predictors of creativity. Intrinsic/extrinsic motivation is one of the most basic distinctions in the field of psychology; the former refers to doing something for the sake of personal interest and enjoyment, while the latter refers to doing something for a reward or some kind of attractive outcome (Deci, 1975; Collins and Amabile, 1999). The relationship between intrinsic/extrinsic motivation and creativity has been of interest to researchers since the 1990s. The literature has consistently shown that intrinsic motivation is conducive to creativity in both Western and Eastern populations (Amabile, 1983; Dong, 1993; Yang and Zhang, 2004; Cooper and Jayatilaka, 2006; Prabhu et al., 2008). When driven by personal interest in a task, individuals are more likely to spend considerable time and effort exploring various or new ways to complete the task and are less likely to accomplish it through the most direct and typical means. Therefore, intrinsic motivation is more conducive to creativity. Regarding the relationship between extrinsic motivation and creativity, previous studies obtained inconclusive results. Some studies have reported that creativity might be reduced by rewards (Amabile, 1983; Dong, 1993; Yang and Zhang, 2004), whereas other studies have suggested that extrinsic motivation could be conducive to creativity (Collins and Amabile, 1999; Eisenberger and Rhoades, 2001; Xue et al., 2001; Cooper and Jayatilaka, 2006). In addition, some studies have reported that there was no significant relation between extrinsic motivation and creativity (Guo et al., 2000; Tang et al., 2015). More recently, Amabile and Pratt (2016) brought about the concept of synergistic extrinsic motivation and insisted that some kinds of extrinsic motivators can show a positive effect on intrinsic motivation and creativity. Thus, not only is intrinsic motivation conducive to creativity, but informational or enabling extrinsic motivation can also be conducive to creativity, especially if initial levels of intrinsic motivation are high; however, controlling extrinsic motivation is detrimental to creativity (Amabile and Pratt, 2016). Similarly, Fischer et al. (2019) also revealed that extrinsic motivators such as relational rewards may have a positive effect on intrinsic motivation and creativity, while extrinsic motivators such as transactional rewards may have no effect on intrinsic motivation and creativity. Therefore, intrinsic motivation shows a positive effect on creativity, while the effect of extrinsic motivation is far more complex.

The previous literature has also revealed a close relationship between regulatory focus and intrinsic/extrinsic motivation. For example, Smith et al. (2009) found that promotion focus could increase intrinsic motivation; specifically, promotion-focused individuals not only view a boring task as more interesting but also put forth the effort to make it more interesting. Similarly, Li et al. (2016) argued that promotion-focused persons prefer tactics that produce desirable results, and they are more likely to be driven by autonomous motivation, which is closer to the intrinsic motivation end of the self-determination continuum of Deci and Ryan (1985); in contrast, prevention-focused persons prefer tactics that allow them to avoid undesirable results, and they are more likely to be driven by controlling motivation, which is closer to the extrinsic motivation end of the aforementioned self-determination continuum. Furthermore, Vaughn’s recent need-support model (Vaughn, 2016a) emphasized the close relation between self-determination theory (Deci and Ryan, 2000) and regulatory focus theory (Higgins, 1997, 1998). Vaughn (2016b) found that when pursuing promotion-focused goals, people experience more intrinsic motivation and identified motivation (“somewhat intrinsic motivation,” Ryan and Deci, 2000), whereas when pursuing prevention-focused goals, they experience more extrinsic motivation and introjected motivation (“somewhat extrinsic motivation,” Ryan and Deci, 2000). In addition, Vaughn (2016a) reported that compared with prevention focus, promotion focus leads to higher satisfaction of autonomy, competence, and relatedness needs; according to the self-determination theory (Deci and Ryan, 2000; Ryan and Deci, 2000; Vansteenkiste and Ryan, 2013), high satisfaction of psychological needs promotes intrinsic motivation. Thus, promotion focus might indirectly improve intrinsic motivation through the satisfaction of autonomy, competence, and relatedness needs. In short, promotion focus shows a closer association with intrinsic motivation than prevention focus.

Regarding the relationship between regulatory focus and creativity, there have been some studies on the underlying mechanism. For example, Baas et al. (2008) found that the interaction between mood activation level and its associated regulatory focus predicts creativity; specifically, high-activating and promotion-focused mood improves creativity, while high-activating and prevention-focused mood inhibits creativity. Further research (Baas et al., 2011) revealed that regulatory closure (whether the goal is attained or not) may moderate the relation between regulatory focus and creativity. Indeed, only in the closure-present condition (goal attained) did the promotion focus group show higher creativity than the prevention focus group, while no difference was found between two foci in the closure-absent condition (goal not attained); furthermore, the activation level mediated the relation between the closure × focus interaction and creativity.

However, questions remain. First, some of these studies (for example, Baas et al., 2008) focused on the relation between mood and creativity, viewing the regulatory focus as a moderator or mediator variable between them, rather than focused on the relation between regulatory focus and creativity. Second, previous literature has investigated the moderating effect underlying the regulatory focus-creativity link but has not probed the mediating variable between regulatory focus and creativity; thus, we know little about the path through which regulatory focus influences creativity. Third, previous findings on the relation between regulatory focus and creativity were mainly obtained among undergraduate students [the only exception being the research of Jin et al. (2016)]; thus, it is not clear whether the conclusions can be generalized to other age groups such as high school students, who are in the critical developmental period for thinking and creativity.

Therefore, the present study aimed to examine the relationship between regulatory focus and creativity in conjunction with intrinsic/extrinsic motivation among high school students, with a focus on whether intrinsic/extrinsic motivation mediates the relationship between regulatory focus and creativity. Based on the aforementioned close relationship among regulatory focus, intrinsic/extrinsic motivation, and creativity, we proposed the following hypotheses:

**Hypothesis 1**: Promotion focus is positively related to creativity, while prevention focus is negatively related to creativity.
**Hypothesis 2**: Intrinsic motivation is positively correlated with creativity, while extrinsic motivation is negatively correlated with creativity.
**Hypothesis 3**: Intrinsic motivation is positively correlated with promotion focus and negatively correlated with prevention focus, while extrinsic motivation is positively correlated with prevention focus and negatively correlated with promotion focus.
**Hypothesis 4**: Regulatory focus predicts creativity through the mediation of intrinsic/extrinsic motivation.

## Materials and Methods

### Participants and Procedure

A total of 418 senior high school students (43% male; *M*_age_ = 16.26, *SD* = 0.67, range = 15–18 years) in Beijing, China, participated in this study. Among the participants, 54% were in senior grade 7 (*n* = 224), and 46% were in senior grade 8 (*n* = 194). The survey was administered in a classroom setting. The participants completed the Regulatory Focus Questionnaire, the Working Preference Inventory, the Williams Creativity Assessment Packet, and the Kirton Adaption–Innovation Inventory successively. All participants were given the option to not participate in the survey and to withdraw from the survey at any time without consequence. After finishing all the questionnaires, they were thanked and compensated with a stationery set valued at approximately ¥10 (approximately $1.40).

### Instruments

#### Regulatory Focus Questionnaire

The Regulatory Focus Questionnaire (Higgins et al., 2001) consists of a promotion focus subscale (six items; e.g., “How often have you accomplished things that got you “psyched” to work even harder?”) and a prevention focus subscale (five items; e.g., “Did you get on your parents’ nerves often when you were growing up?”). The participants responded on a five-point Likert scale ranging from 1 (*never*) to 5 (*very often*). In this study, the alphas of the two subscales were 0.73 and 0.72.

#### Working Preference Inventory

The Working Preference Inventory (Amabile et al., 1994) consists of an intrinsic motivation subscale (15 items; e.g., “I want to find out how good I really can be at my work”) and an extrinsic motivation subscale (15 items; e.g., “I am strongly motivated by the recognition I can earn from other people”). The participants indicated their agreement or disagreement on a five-point Likert scale ranging from 1 (*strongly disagree*) to 5 (*strongly agree*). In this study, the alphas of the intrinsic motivation and extrinsic motivation subscales were 0.75 and 0.70, respectively.

#### Williams Creativity Assessment Packet

The Williams Creativity Assessment Packet (Chinese version, Lin and Wang, 1997) consists of four dimensions: curiosity (14 items; e.g., “I like to do a lot of new things”), complexity (12 items; e.g., “I often do things in the same way, and I don’t like to look for other new methods”), risk-taking (11 items; e.g., “It’s fun to try new games and activities”), and imagination (13 items; e.g., “I like to imagine that I will become an artist, a musician, or a poet someday”). The participants indicated their choices on a three-point Likert scale ranging from 1 (*certainly false*) to 3 (*certainly true*). In this study, the total alpha was 0.86.

#### The Kirton Adaption–Innovation Inventory

The Kirton Adaption–Innovation Inventory (Kirton, 1976) consists of three subscales: originality (13 items; e.g., “Copes with several new ideas at the same time”), efficiency (seven items; e.g., “Is methodical and systematic”), and rule (12 items; e.g., “Never seeks to bend or break the rules”). The participants responded on a five-point Likert scale ranging from 1 (*certainly false*) to 5 (*certainly true*). In this study, the alphas of the three subscales were 0.77, 0.74, and 0.75, respectively.

Since creativity comprises different aspects, such as creative thinking aspects (i.e., cognitive aspects of creative information processing) and creative personality aspects (i.e., the personal inclinations that are vital to creative activities) (Zhang et al., 2007), two measures were combined in the present study, the Williams Creativity Assessment Packet score was the indicator of creative personality, and the Kirton Adaption–Innovation Inventory score was the indicator of innovative-adaptive cognitive style.

## Results

### Common Method Bias Test

We adopted Harman’s single-factor analysis to examine possible common method bias. The results suggested that there were 27 factors with characteristic roots greater than 1, with a total contribution rate of 62.59%; the variation contribution rate of the first factor was 11.95%. Thus, there was no severe common method bias in this study.

### Difference Tests

A *t*-test showed that there was a significant gender difference in prevention focus (*t* = 2.51, *p* = 0.01, Cohen’s *d* = 0.25) and that female participants (*M* = 3.57, *SD* = 0.65) were more prevention focused than were male participants (*M* = 3.40, *SD* = 0.66). Moreover, there was a significant grade difference in intrinsic motivation (*t* = 2.102, *p* = 0.036, Cohen’s *d* = 0.21); the participants in grade 7 (*M* = 3.80, *SD* = 0.45) were more intrinsically motivated than those in grade 8 (*M* = 3.70, *SD* = 0.46).

### Correlation Analysis

Table 1 shows the descriptive statistics and the correlation coefficients between variables. Intrinsic motivation and promotion focus were both positively correlated with two indicators of creativity, and extrinsic motivation and prevention focus were both negatively correlated with adaptive–innovative cognitive style. In addition, promotion focus and prevention focus were both positively correlated with intrinsic motivation; however, neither type of focus was significantly correlated with extrinsic motivation.

**TABLE 1:** Means, standard deviations, and correlations for the observed variables.

	*M*	*SD*	Promotion	Prevention	Intrinsic	Extrinsic	Creative	Innovative–adaptive
			focus	focus	motivation	motivation	personality	cognitive style
Promotion focus	3.38	0.53	1					
Prevention focus	3.49	0.67	0.32**	1				
Intrinsic motivation	3.75	0.46	0.41**	0.20**	1			
Extrinsic motivation	3.29	0.54	−0.16	−0.04	−0.02	1		
Creative personality	9.06	1.01	0.31**	0.04	0.50**	0.05	1	
Innovative–adaptive cognitive style	98.59	11.44	0.13*	−0.24**	0.32**	−0.27**	0.34**	1

**p < 0.01, *p < 0.05.

### Measurement Model

Confirmatory factor analysis was conducted for promotion focus, prevention focus, intrinsic motivation, extrinsic motivation, and creativity. The item parceling method was used for the former four variables, in which we used a factorial algorithm (Rogers and Schmitt, 2004) to create three parcels with three to five items in each parcel, and creativity was measured by the Williams Creativity Assessment Packet score and Kirton Adaption–Innovation Inventory score. The model fits the data moderately well, χ^2^ (58, *N* = 418) = 126.95, CFI = 0.95, TLI = 0.92, and RMSEA = 0.053. All indicators loaded significantly (*ps* < 0.001) on each latent variable, and the standardized factor loadings were between 0.33 and 0.85.

### Path Model

**FIGURE 1:**
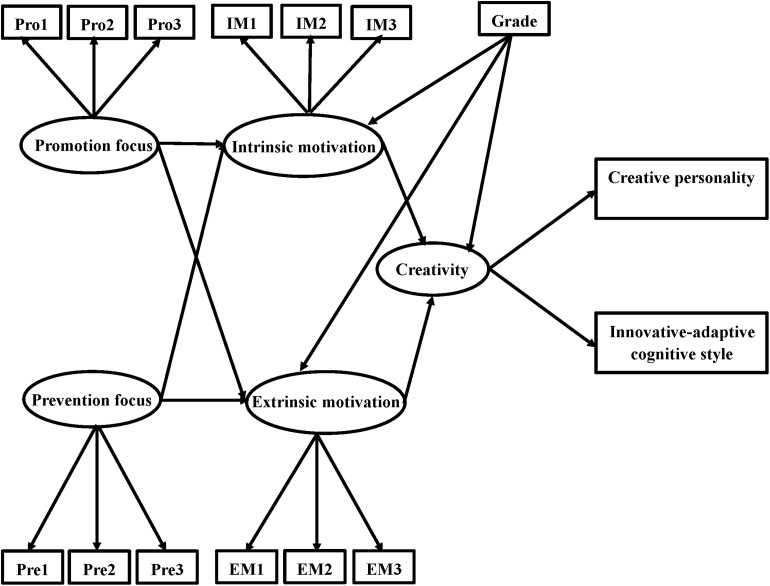
The hypothesized model of regulatory focus, motivation, and creativity.

Based on the aforementioned hypotheses, we proposed the hypothesized model depicted in Figure 1, in which promotion focus and prevention focus both predicted creativity through the mediation of intrinsic motivation and extrinsic motivation, and the effect of grade was controlled because of the significant difference in intrinsic motivation between grades.

**FIGURE 2:**
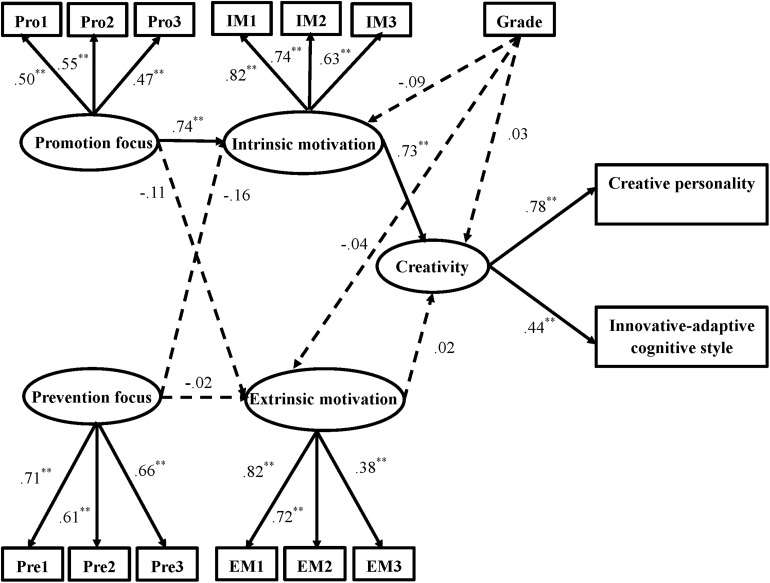
The hypothesized model of regulatory focus, motivation, and creativity with path coefficients. **p < 0.01; ⇢: indicates path that was hypothesized but not significant.

The hypothesized model was tested using structural equation modeling (SEM) with Mplus. The SEM results revealed that the fit was inadequate, χ^2^ (81, *N* = 418) = 311.71, *p* < 0.01, CFI = 0.83, TLI = 0.78, RMSEA = 0.083. In addition, as shown in Figure 2, some paths in the hypothesized model were not significant. Both the fit indexes and path coefficients indicated that further adjustment of the model was necessary.

**FIGURE 3:**
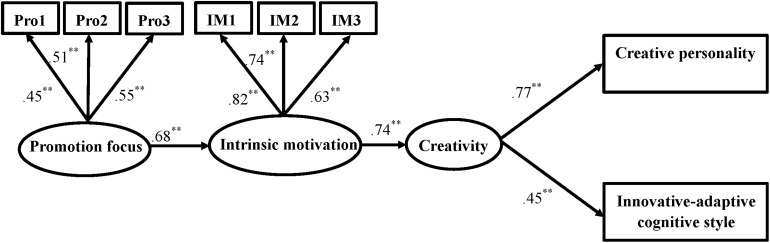
The modified model of regulatory focus, motivation, and creativity. **p < 0.01.

The hypothesized model was adjusted according to the modification indexes, and all the non-significant paths were removed from the model, i.e., the paths from grade to intrinsic motivation, extrinsic motivation, and creativity, the path from prevention focus to extrinsic motivation, the path from prevention focus to intrinsic motivation, the path from promotion focus to extrinsic motivation, and the path from extrinsic motivation to creativity. Thus, we obtained the modified model shown in Figure 3. The modified model fits the data well, χ^2^ (18, *N* = 418) = 43.40, *p* < 0.01, CFI = 0.96, TLI = 0.94, and RMSEA = 0.058.

The modified model indicated that promotion focus positively predicted intrinsic motivation, which, in turn, positively predicted creativity. Thus, taken together, the results show that promotion focus positively predicted creativity through the mediation of intrinsic motivation. Moreover, we examined the mediating role of intrinsic motivation by calculating the 95% confidence interval (CI) of the effects of promotion focus on creativity through bootstrapping with 1,000 random samples. The indirect effect of promotion focus on creativity was 0.50, and the 95% CI did not include zero (95% CI = [0.361, 0.648]), which suggests that the mediating effect was significant.

## Discussion

### Gender Differences on Regulatory Focus

In our study, female participants were more prevention focused than were male participants. Higgins (1997) argued that regulatory focus is closely related to parenting behavior; if parents prefer the satisfaction of growth needs, children are more likely to become promotion focused, whereas if parents prefer the satisfaction of security needs, children tend to become prevention focused. In China, parents traditionally show differential rearing styles for daughters and sons (Xu, 2008; Zeng, 2010). Specifically, parents are more concerned with the satisfaction of the growth needs of sons, encouraging boys’ exploration and innovation; in contrast, they are more concerned with the satisfaction of the security needs of daughters, guiding girls to follow rules and to protect their personal safety (Xu, 2008). As a result, in Chinese participants, girls might be more prevention focused than boys.

### The Relationship Between Regulatory Focus and Creativity

Our study revealed a positive association between promotion focus and creativity, and this result is consistent with previous research (e.g., Friedman and Förster, 2001; Lam and Chiu, 2002; Jin et al., 2016; Lin, 2017). The reason for this finding may be that promotion-focused individuals are inclined to explore more strategies to solve problems, which will result in the generation of more creative ideas (Lam and Chiu, 2002). In addition, promotion focus is closely related to an integrated, global information processing style, which helps activate more cognitive elements and further benefits the establishment of more and unusual connections among cognitive elements (Semin et al., 2005; Sacramento et al., 2013). Furthermore, a previous study indicated that people who are promotion focused are more inclined to invest in risk-taking behavior than those who are prevention focused (Hamstra et al., 2011). Sacramento et al. (2013) also reported that people who are promotion focused are more likely to choose a risky scheme, which, in turn, enhances creativity, whereas people who are prevention focused tend to choose safe and conservative regimes, which inhibits creativity. Additionally, regulatory focus was closely related to openness. For example, studies have found that openness is positively associated with promotion focus and negatively connected with prevention focus because promotion-focused individuals are less risk averse and enjoy changing and exploring the world, whereas people who are prevention focused are inclined to follow the rules and avoid unknown or unpredictable results (Vaughn et al., 2008; Yen et al., 2011; Lanaj et al., 2012).

On the other hand, in our study, regarding the relation between prevention focus and creativity, SEM revealed no significant results. In the previous literature, some studies have supported the negative effect of prevention focus on creativity (Baas et al., 2008; Bittner and Heidemeier, 2013); however, inconsistent results have been reported. For example, a meta-analysis revealed that prevention-focused states lead to similar levels of creativity as promotion-focused states if the task goal has not been reached (Baas et al., 2011). Another study suggested that regulatory focus and the evaluation subtype interact to influence creativity; promotion focus might improve creativity in information evaluation situations, whereas prevention focus might benefit creativity in controlling evaluation situations (Wang et al., 2017). In addition, the effect of regulatory focus on creativity might depend on situational factors, such as task demand (Li, 2015); promotion focus might benefit creativity under high task demand, while prevention focus might benefit creativity under low task demand. Thus, although researchers have argued that prevention focus might be harmful to creativity due to the associated sensitivity to negative outcomes (Lam and Chiu, 2002), the preference for vigilant strategies (Sacramento et al., 2013), and the preference for a local processing style (Semin et al., 2005; Sacramento et al., 2013), the effects of prevention focus are more complex than the effects of promotion focus and should be discussed in greater detail in future research.

### The Relationship Between Intrinsic/Extrinsic Motivation and Creativity

Our study showed that intrinsic motivation was positively associated with creativity, and this result is consistent with previous studies (Amabile, 1983; Dong, 1993; Yang and Zhang, 2004). Higher intrinsic motivation can expand an individual’s attention and cognition and stimulate associations between ideas, which is beneficial to fluency and the flexibility of ideas (Grant and Berry, 2011). In addition, researchers have argued that individuals with high intrinsic motivation are more inquisitive, adventurous, and cognitively flexible and show high persistence in the face of obstacles (Shalley et al., 2004); therefore, intrinsic motivation facilitates the production of original and unusual thoughts.

However, regarding the relationship between extrinsic motivation and creativity, SEM showed that there was no significant path between them, and this result is consistent with studies by Guo et al. (2000) and Tang et al. (2015). As mentioned in the “Introduction” section, the existing literature on the relationship between extrinsic motivation and creativity has not reached a consistent conclusion, and positive, negative, and non-significant results have been reported. We postulate that both the inconsistency of the results and the non-significant results may be explained by the existence and effect of some moderator variables. Research has suggested that the relationship between competitiveness level and creativity differs between a closed group (whose members do not change) and an open group (in which new members join and original members leave) (Baer et al., 2010). Conti et al. (2001) also found that competition had different effects on males and females, improving creativity for boys but reducing creativity for girls, because boys view competition as challenging and exciting, but girls feel controlled under conditions of competition (Conti et al., 2001). More closely related to our study, recent work by Amabile and Pratt (2016) suggested that synergistic extrinsic motivation can be conducive to creativity, while controlling extrinsic motivation would undermine creativity. Fischer et al. (2019) also revealed that relational rewards and transactional rewards show different effects on intrinsic motivation and creativity. Earlier studies of Ryan et al. (1983) also found that controlling extrinsic motivation inhibits intrinsic motivation, while informational extrinsic motivation improves intrinsic motivation. Thus, the relationship between extrinsic motivation and creativity may depend on the different types of extrinsic motivation. In our study, the items in the instruments of extrinsic motivation involve competition, evaluation, recognition, money or other tangible incentives, and constraint by others (Amabile et al., 1994), which means that the extrinsic motivation measured by it was a composition of synergistic extrinsic motivation, informational extrinsic motivation, and controlling extrinsic motivation, and this may be the reason why in our study there was no significant relationship between extrinsic motivation and creativity.

### The Relationship Between Promotion Focus and Intrinsic Motivation

Our study also found that promotion focus was positively associated with intrinsic motivation, and this result is consistent with previous studies (Deci and Ryan, 2000; Smith et al., 2009; Vansteenkiste and Ryan, 2013; Li et al., 2016; Vaughn, 2016a,b). Previous research has suggested that regulatory focus affects intrinsic motivation both directly and indirectly. Promotion-focused individuals not only experience higher interest in a boring task but also try to make the task more interesting (Smith et al., 2009). Vaughn’s (2016a,b, 2019) recent work revealed that people who pursue promotion-focused goals experience more intrinsic motivation, while people who pursue prevention-focused goals experience more extrinsic motivation, which might be because a promotion focus leads to higher satisfaction of autonomy, competence, and relatedness needs than prevention focus. Therefore, promotion focus might improve intrinsic motivation through the satisfaction of autonomy, competence, and relatedness needs.

### The Mediating Effect of Intrinsic Motivation Between Regulatory Focus and Creativity

Moreover, consistent with our hypotheses, our study supported the mediating effect of intrinsic motivation between regulatory focus and creativity. This finding might be explained by the beneficial effect of intrinsic motivation on creativity (Amabile, 1983) and Vaughn’s need-support model (Vaughn, 2016a); the latter combines regulatory focus theory (Higgins, 1997, 1998) and self-determination theory (Deci and Ryan, 2000; Ryan and Deci, 2000) to reveal the relation between regulatory focus and intrinsic/extrinsic motivation. Vaughn’s need-support model (Vaughn, 2016a) suggests that promotion focus might result in intrinsic motivation; furthermore, numerous studies have supported that intrinsic motivation is conducive to creativity (Amabile, 1983; Dong, 1993; Yang and Zhang, 2004; Cooper and Jayatilaka, 2006; Prabhu et al., 2008). Thus, promotion focus might benefit creativity by improving intrinsic motivation.

## Contributions

Regarding the relationship between regulatory focus and creativity, there have been some studies on the underlying moderating mechanism (for example, Baas et al., 2008, 2011); however, previous literature has not probed the mediating effect underlying the regulatory focus–creativity link. Thus, since our study revealed the mediating role of intrinsic motivation, it contributed to the existing literature by enlightening the understanding of the path through which regulatory focus exhibits its impact on creativity, and providing and initiating a new research perspective on the regulatory focus–creativity link. Second, Vaughn’s (2016a,b, 2019) need-support model emphasized the close relationship between regulatory focus and intrinsic/extrinsic motivation; thus, our finding about the mediating role of intrinsic motivation in the regulatory focus–creativity link provided empirical support for the need-support model. Third, previous studies on the relationship between regulatory focus and creativity focused mainly on undergraduate students [with the exception of the research of Jin et al. (2016)]; thus, our study focusing on adolescent participants greatly enriched the previous literature by improving the understanding of adolescents’ creativity development and its determinants.

The results of our study also have practical implications for the cultivation of creativity in the settings of school and family. Although creative thinking training is important and effective for creativity cultivation (Shen et al., 2000; Sun et al., 2016; Kashani-Vahid et al., 2017), it is not the only approach, since creativity involves both an ability/thinking aspect and a motivation/attitude aspect (Wang et al., 1998; Sternberg, 1999; Li and Zhang, 2006). Thus, from this perspective, our study underlined the importance of the motivation approach of creativity cultivation.

Moreover, given that promotion focus is beneficial to creativity, fostering promotion focus may result in the improvement of creativity. Higgins (1997) asserted that regulatory focus is closely related to parenting behavior, especially how parents satisfy children’s needs; to be detailed, the satisfaction of children’s growth needs would strengthen promotion focus, while the satisfaction of children’s safety needs would induce prevention focus. Thus, to instill promotion focus, parents should pay more attention and provide enough satisfaction to children’s growth needs rather than safety needs, encourage their exploration to the world, and provide necessary assistance for them. This is especially of great importance for girls because they are more prevention focused than boys. Parents of girls should deliberately reduce the various restraints imposed on girls for the sake of safety and avoid delivering high-level worry about safety to them. Furthermore, regulatory focus is not only a chronic proposition resulting from long-term family environmental influences but also an inclination that is sensitive to temporary situational induction (Zaal et al., 2011). Thus, in the school setting, to induce promotion focus, teachers can guide students to pay more attention to their personal interests, aspirations, and ideals rather than duties and responsibilities.

In addition, since intrinsic motivation is conducive to creativity, the enhancement of intrinsic motivation is also an important path of creativity cultivation. According to the self-determination theory (Deci and Ryan, 2000), autonomy support from teachers or parents can provide satisfaction to students’ psychological needs, which would further induce intrinsic motivation; in contrast, environment control can result in the frustration of psychological needs, which would further induce extrinsic motivation (Costa et al., 2016; Collie et al., 2019). Thus, teachers and parents should create a more autonomy-supportive atmosphere instead of an atmosphere full of stress, threat, and strict constraints. In the classroom, teachers can guide students to set appropriate goals based on their personal interests and innovate teaching methods that incentivize students’ curiosity, which are also helpful to instill students’ intrinsic motivation.

## Limitations and Future Directions

Some limitations of this study must be addressed. First, since the data used in this study are cross-sectional, the conclusions on the relationships between variables must be treated with caution. Future research can further examine this relationship with an experimental or longitudinal research design. Second, in our study, the hypothesized mediating effect of extrinsic motivation was not supported, and extrinsic motivation showed no significant relationship with regulatory focus and creativity. Although there is a possibility that this result is valid, there might be other possibilities. For example, this result might imply the existence of a floor effect, i.e., the extrinsic motivation scores were too low, and therefore, the relation between extrinsic motivation and other variables was concealed. In addition, this result might be associated with the measure of extrinsic motivation. In our study, extrinsic motivation was measured by the extrinsic motivation subscale of the Working Preference Inventory (Amabile et al., 1994), in which the items involve synergistic extrinsic motivation, informational extrinsic motivation, and controlling extrinsic motivation; thus, this compound extrinsic motivation would show no effect on creativity, since different types of extrinsic motivation exhibit different effects on creativity (Amabile and Pratt, 2016). Future research can further explore the extrinsic motivation–creativity link by adopting instruments that can assess different types of extrinsic motivation. Last, our study found no significant relationship between prevention focus and creativity. Future research using alternative measures of both regulatory focus and creativity (such as creative tasks or behavior indexes) may reveal a clearer relationship between prevention focus and creativity, as well as the underlying mediation mechanism and moderation mechanism between them.

## Conclusion

In this study, SEM showed that promotion focus positively predicted intrinsic motivation, which, in turn, positively predicted creativity, and the bootstrapping method showed that the mediating effect was significant. In general, our study suggested that intrinsic motivation played a mediating role between promotion focus and creativity in adolescents.

## Data Availability Statement

The raw data supporting the conclusions of this article will be made available by the authors, without undue reservation.

## Ethics Statement

The studies involving human participants were reviewed and approved by Ethics Committee of Psychology College of Capital Normal University. Written informed consent to participate in this study was provided by the participants’ legal guardian/next of kin.

## Author Contributions

LW contributed to the conception and design of the study. JW contributed to the participants recruit and organized the survey. XW and KD organized the database. YC, XW, and ZL performed the statistical analysis. XW wrote the first draft of the manuscript. LW, XW, and YC wrote the sections of the manuscript.

## Conflict of Interest

The authors declare that the research was conducted in the absence of any commercial or financial relationships that could be construed as a potential conflict of interest.
